# Interventions and implementation considerations for reducing pre-treatment loss to follow-up in adults with pulmonary tuberculosis: A scoping review

**DOI:** 10.12688/f1000research.157439.1

**Published:** 2024-11-27

**Authors:** Mercy Mulaku, Eddy Johnson Owino, Eleanor Ochodo, Taryn Young

**Affiliations:** 1 Department of Malaria, Centre for Global Health Research, Kenya Medical Research Institute, Kisumu, Kenya; 2 Faculty of Medicine and Health Sciences, Division of Epidemiology and Biostatistics, Stellenbosch University Centre for Evidence-Based Health Care, Cape Town, South Africa; 3 Department of Pharmacology, Clinical Pharmacy and Pharmacy Practice, University of Nairobi Faculty of Health Sciences, Nairobi, Kenya

**Keywords:** Tuberculosis, interventions, pre-treatment loss to follow-up, implementation, scoping review

## Abstract

**Background:**

Tuberculosis (TB) is a leading cause of death worldwide with over 90% of reported cases occurring in low- and middle-income countries (LMICs). Pre-treatment loss to follow-up (PTLFU) is a key contributor to TB mortality and infection transmission.

**Objectives:**

We performed a scoping review to map available evidence on interventions to reduce PTLFU in adults with pulmonary TB, identify gaps in existing knowledge, and develop a conceptual framework to guide intervention implementation.

**Methods:**

We searched eight electronic databases up to February 6 2024, medRxiv for pre-prints, and reference lists of included studies. Two review authors independently selected studies and extracted data using a predesigned form. We analysed data descriptively, presented findings in a narrative summary and developed a conceptual framework based on the Practical, Robust Implementation, and Sustainability Model to map the factors for effective intervention implementation.

**Results:**

We reviewed 1262 records and included 17 studies. Most studies were randomized controlled trials (8/17, 47%). Intervention barriers included stigma and inadequate resources; enablers included mobile phones and TB testing and results on the same day. We identified eight interventions that reduced PTLFU: treatment support groups; mobile notifications; community health workers; integrated HIV/TB services; Xpert MTB/RIF as the initial diagnostic test; computer-aided detection with chest radiography screening; active linkage to care; and multi-component strategies.

**Conclusion:**

Given the variation of healthcare settings, TB programs should consider contextual factors such as user acceptability, political commitment, resources, and infrastructure before adopting an intervention. Future research should utilize qualitative study designs, be people-centred, and include social and economic factors affecting PTLFU.

**List of abbreviations**
CHW: Community Health Worker
CINAHL: Cumulative Index to Nursing and Allied Health Literature
DCXR-CAD
: Computer-aided digital chest X-ray
HCW: Health Care Worker
HERDIN: Health Research and Development Information Network
LILACS: Latin American and Caribbean Health Sciences
mWRD: Molecular WHO-recommended rapid diagnostics
OSF: Open Science Framework
PCC: Population, Concept, Context framework
POC: Point of care
PRISM: Practical Robust Implementation and Sustainability Model
PRISMA-P
: Preferred Reporting Items for Systematic reviews and Meta-Analyses-Protocol
PRISMA-ScR
: Preferred Reporting Items for Systematic Reviews and Meta-analysis extension for scoping review
PTLFU: Pre-treatment Loss To Follow-Up
RE-AIM
: Reach, Effectiveness, Adoption, Implementation, Maintenance implementation framework
SMS: Short Message Service
SoC: Standard of care
TB: Tuberculosis
TRIP: Turning Research into Practice
TSG: Treatment support group
WHO: World Health Organization

## Introduction

Tuberculosis (TB) is a leading cause of morbidity and mortality worldwide. In 2021, about 10.6 million people fell ill with TB and 1.6 million people died, including 1.4 million HIV-negative people and 187,000 HIV-positive people. ^1^ Worldwide, from 2015 to 2021, the cumulative reduction in TB incidence was 10% and in mortality was 5.9%, falling short of the goals set by the World Health Organization (WHO) End TB strategy for 2020 for a reduction in TB incidence of 20% and mortality of 35%. ^1, 2^ The COVID-19 pandemic was in part responsible for the limited progress in reaching these goals because of reduced access to essential services for TB diagnosis and treatment. Consequently, for the first time in a decade, TB deaths increased. ^1^

Timely initiation of effective TB treatment is one of the key strategies to reduce mortality and transmission of TB in the community. ^3– 5^ However, delays in the diagnosis and treatment of TB persist. Tragically, people who are diagnosed with TB may never start treatment at all, referred to as ‘pre-treatment loss to follow-up’ (PTLFU). When people who experience PTLFU return to the community, they may spread TB bacteria to others and eventually die. ^6, 7^ A systematic review including 23 studies from 14 countries, eight of which were in Africa, found a high proportion (up to 38%) of PTLFU in Africa. ^8^

Findings from quantitative and qualitative studies have identified several factors that contribute to PTLFU. Some of the challenges highlighted by these studies are misconceptions about TB and its management, stigma, lack of psychosocial support, unclear communication with patients on steps to take after testing positive for TB, dissatisfaction with clinic wait times, incomplete patient contact information, and human resource and financial constraints faced by healthcare workers (HCWs). ^8, 9^

Several studies have pointed out various interventions to reduce loss to follow-up in different chronic conditions such as the use of patient navigators (i.e., people who help patients reach the next steps in care), ^10, 11^ community health extension workers, ^12^ mobile phone reminders, and electronic patient tracking tools. In a systematic review (including 25 studies), Law and colleagues found that psychosocial support was key to reducing loss to follow-up in persons with drug-resistant TB. ^13^ None of these interventions were directed at PTLFU in people with pulmonary TB. To our knowledge, a conceptual framework for the implementation of interventions for PTLFU has not been described.

We performed a scoping review to identify and map the available evidence on interventions to reduce PTLFU in adults with pulmonary TB. In addition, we summarized the effects of these interventions on PTLFU and described barriers and enablers to implementing it. From this, we developed a conceptual framework adapted from the Practical, Robust Implementation, and Sustainability model to guide the implementation of the identified interventions. ^14– 16^

## Methods

We based this scoping review on the framework proposed by Arksey and O’Malley and modified by Levac. ^17– 19^ The stages of the framework are (i) identifying the research question; (ii) identifying relevant studies; (iii) study selection; (iv) charting the data; (v) collating, summarizing, and reporting the results; and (vi) consultation with stakeholders (optional).

### Identifying the research question

The overarching review question was ‘What is the available evidence on interventions that reduce PTLFU in adults with pulmonary TB?’ The specific review questions were as follows.

1. What evidence exists on interventions to reduce PTLFU in adults with pulmonary TB?
2. What are the effects of the interventions in reducing PTLFU in adults with pulmonary TB?
3. What are the barriers and enablers to the implementation of these interventions in adults with pulmonary TB?

*Eligibility criteria*

We used the Population, Concept, Context (PCC) framework to guide our eligibility criteria and study selection according to the research question.

*Population* as studies with the following participant characteristics:

- Adults 18 years and older or as described by authors
- Bacteriologically-confirmed pulmonary TB, i.e., a positive result on sputum smear, sputum culture, or sputum molecular WHO-recommended rapid diagnostic test (mWRD)

*Concept* as interventions to reduce PTLFU, where PTLFU was defined as patients in a national TB care programme who received a diagnosis of TB based on at least one positive sputum smear, culture, or mWRD but did not start TB treatment. In this review, we adopted the definition stated in each study for PTLFU as opposed to a definition with a defined time frame. ^8, 20, 21^ Interventions to reduce PTLFU were categorized into interventions targeted at patient, provider, and healthcare system levels.

*Context* as all settings (rural and urban), all levels of healthcare (primary, secondary, and tertiary), and all countries where interventions had been used to reduce PTLFU in adults with pulmonary TB.

*Types of studies.* We included randomized and non-randomized studies and studies with mixed-method study designs that assessed quantitative data regarding interventions, barriers, and enablers. We excluded studies in children, studies primarily focused on extrapulmonary TB, editorials, and comments.

In this review, we defined a barrier and enabler as a systemic and/or experienced factor that reduces or enhances the potential effects of the intervention respectively. ^22^

### Identifying relevant studies

We performed a comprehensive search in the following electronic databases up to February 6, 2024: Cochrane Library (Issue 2 of 12, February 2024), MEDLINE (Ovid from 1946 to September 21, 2022), EMBASE (from 1947), Science Citation Index-Expanded (Web of Science from 1900), Global Index Medicus (from 1900), TRIP (Turning Research into Practice from 1997), SCOPUS (from 1966), and CINAHL (Cumulative Index to Nursing and Allied Health Literature; EBSCO host from 1937). We also searched medRxiv (from 2019) for pre-prints. We performed the search without date restriction and limited our search to the English language. We also reviewed reference lists of included studies to identify relevant articles. We used the following key search terms: “loss to follow-up”, “LTFU”, “policy”, “interventions”, “strategies”, “adults”, “Pulmonary tuberculosis”, and “Pulmonary TB”. Boolean terms OR, AND, and Medical subject heading (MeSH) terms were used during the search to identify relevant studies. We have provided the full search strategy for Medline (Ovid) in supplementary file 1.

### Study selection

We imported all records identified through database searching into EndNote X9 to remove duplicates ^23^ and then transferred to Covidence for study selection. ^24^ Two review authors (MM and EJO) independently screened titles and abstracts and then assessed full-text articles to come up with the final list of included studies. We resolved disagreements through discussion and, if necessary, consulted a third review author. We reported the results from the screening using the PRISMA-ScR flow diagram. ^25^

### Charting the data

We designed a data extraction form to collect information relevant to the objective of this review. MM and EJO independently piloted the data extraction form with four of the included studies and modified the form, accordingly, based on the pilot results before its final use. MM and EJO independently extracted data from all included studies, resolving any disagreements through discussion. We extracted the following data: author, publication year, study aim, study setting, income category according to the World Bank classification, ^26^ TB burden categories according to the WHO classification for 2021 to 2025, ^27^ study design, participant characteristics, intervention, findings, barriers and enablers in intervention implementation.

### Collating, summarizing, and reporting results

We presented a narrative summary detailing description of the intervention; whether the intervention was directed at factors related to patients, HCWs, or the healthcare system; the effect of the intervention on PTLFU; the settings where the interventions were used; and implementation barriers and enablers. We summarized the key findings in tables. We also pointed out research gaps and described implications for future research.

### Development of the conceptual framework

We adapted the Practical, Robust Implementation and Sustainability Model (PRISM) framework because it encompasses all stages of implementation (planning, implementation, and evaluation) therefore it is comprehensive. ^16, 28, 29^ PRISM considers the intervention, external environment, implementation, and sustainability infrastructure, and recipients and how they relate to influencing the implementation process. Based on the review findings, we grouped the available interventions into three domains: patient-related, healthcare worker-related, and healthcare system-related. ^30^ We then demonstrated associations by integrating the recipients, external environment, implementation, and sustainability infrastructure. We developed the framework through an iterative process and discussion among the review authors. ^31^

### Ethics considerations and reporting

We did not pursue a formal ethical review since we used published data that were publicly available. We published the protocol in the Open Science Framework (OSF). ^32^ We reported the findings of the scoping review using Preferred Reporting Items for Systematic Reviews and Meta-analysis extension for scoping reviews (PRISMA-ScR) guidance. ^25^

## Results

### Study selection

**Figure 1.**
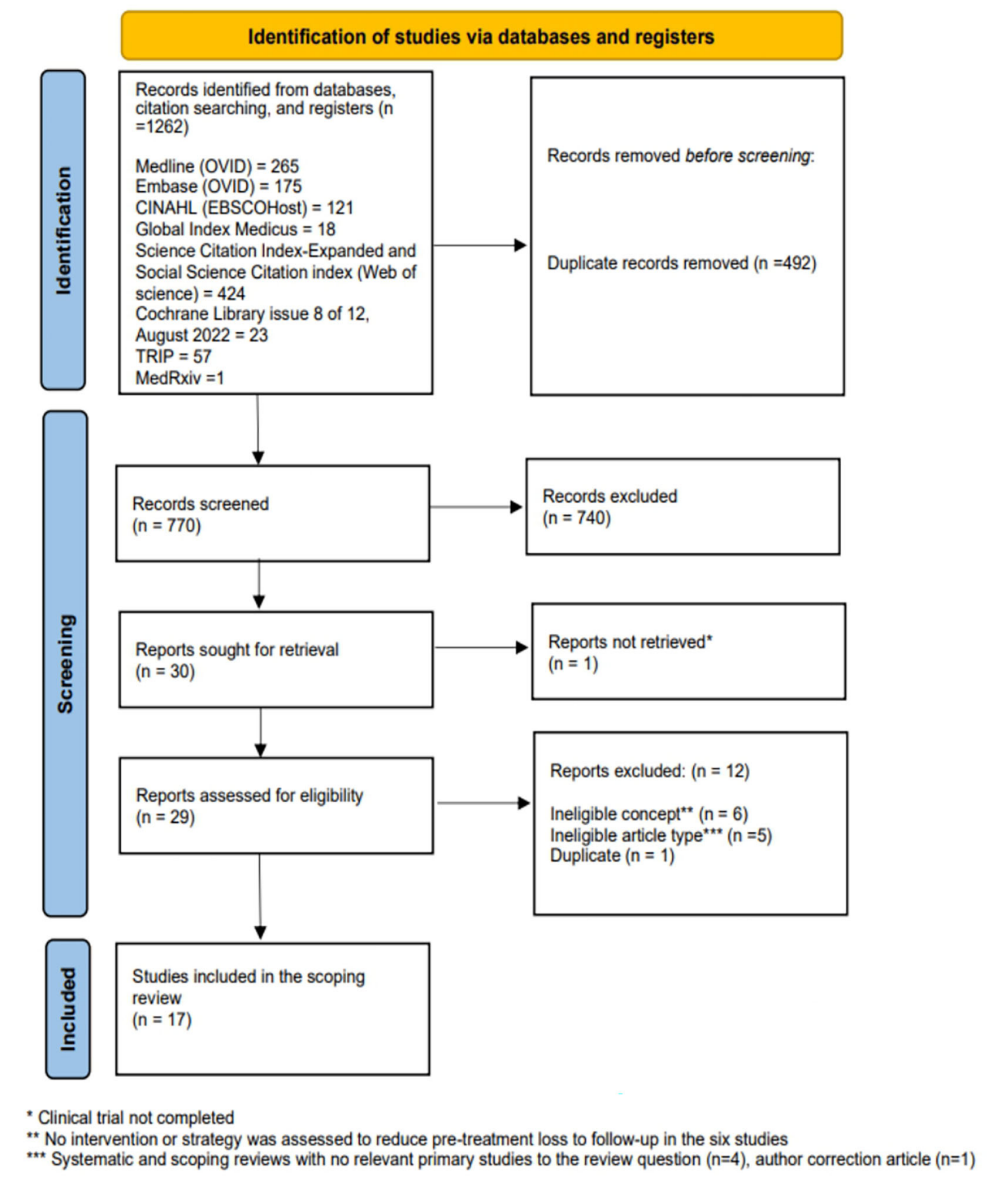
PRISMA-ScR flow diagram of study selection.

We identified 1262 records through electronic database searching, and manual review of reference lists. After removing duplicates, we screened 770 titles and abstracts and excluded 740 irrelevant reports. We retrieved 29 reports for full-text review. We retrieved 29 reports for full-text review, excluded 12 based on eligibility criteria, and included 17 studies in the scoping review ( Figure 1). Details of the excluded studies are in supplementary file 2.

### Characteristics of included studies

We describe key study characteristics in Table 1 (Extended data). Of the 17 included studies, eight (47%) were randomized controlled trials, ^33– 40^ four (24%) were before and after studies ^41– 44^; one (6%) was a community-based intervention ^45^; one (6%) was a situation analysis ^46^; one (6%) was a quasi-experimental study ^47^; one (6%) was a community-based TB prevalence survey ^48^; and one (6%) was a cost and cost-effectiveness analysis. ^49^ One study was a multicentre study, covering four countries in Africa, South Africa, Zambia, Zimbabwe, and Tanzania. ^38^ Considering the multicentre study, in total we had 20 study settings that took place in 8 countries: India (7/20, 35%), South Africa (6/20, 30%), Uganda (2/20, 10%), Malawi (1/20, 5%), Ethiopia (1/20, 5%), Zambia (1/20, 5%), Zimbabwe (1/20,5%), and Tanzania (1/20, 5%). Of the 20 study settings where the studies were conducted, eight (40%) were lower-middle income, seven (35%) were upper-middle income countries, and five (25%) were low-income countries. Nineteen study settings were considered high burden for TB, 20 were high burden for TB/HIV, and 14 were high burden for MDR-TB/rifampicin-resistant TB. The participants were people with TB, HCWs, healthcare managers, and community residents.

### Description of the interventions

**Figure 2.**
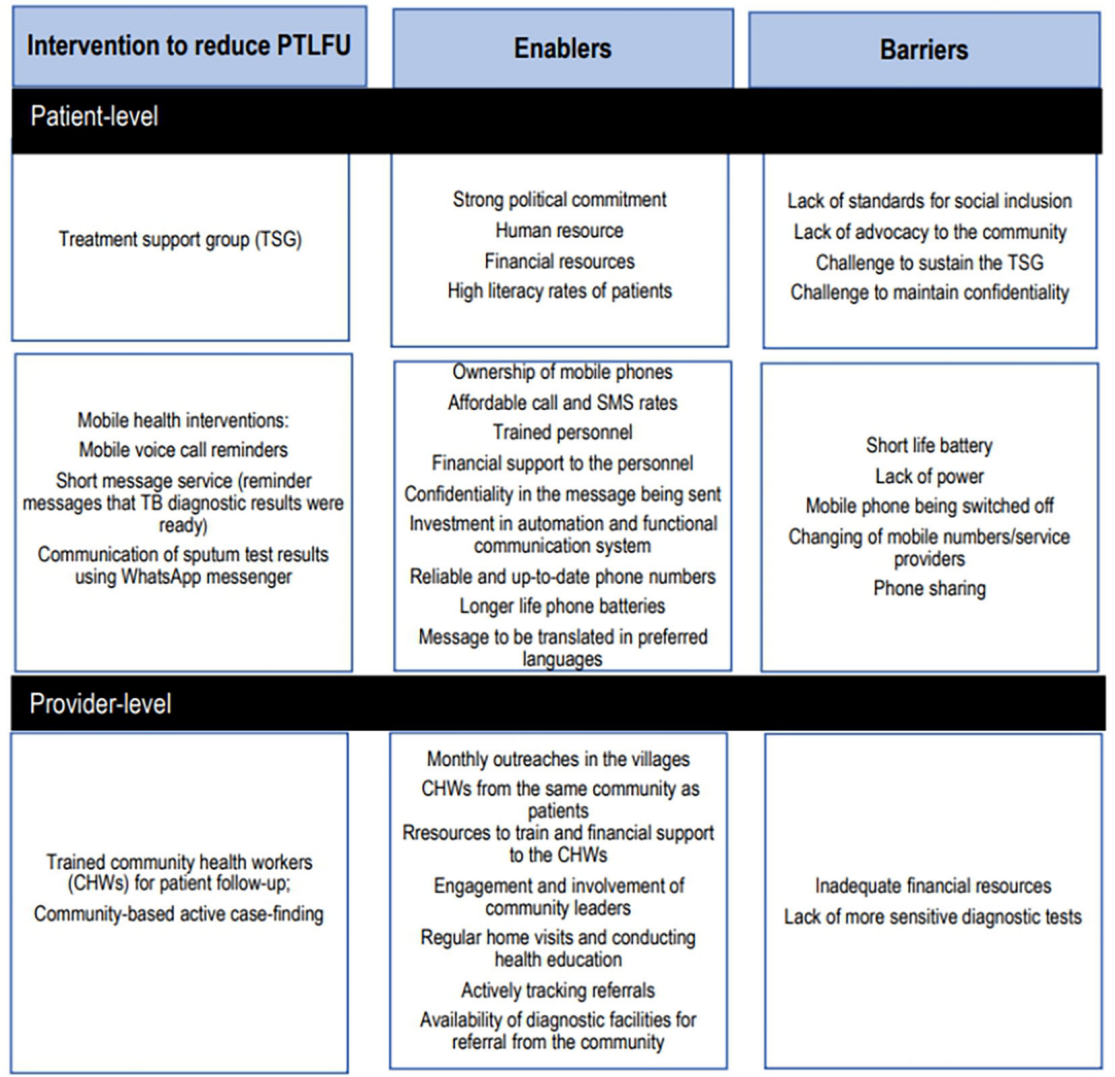
Interventions to reduce pre-treatment loss to follow-up, enablers and barriers at patient and provider levels.

**Figure 3.**
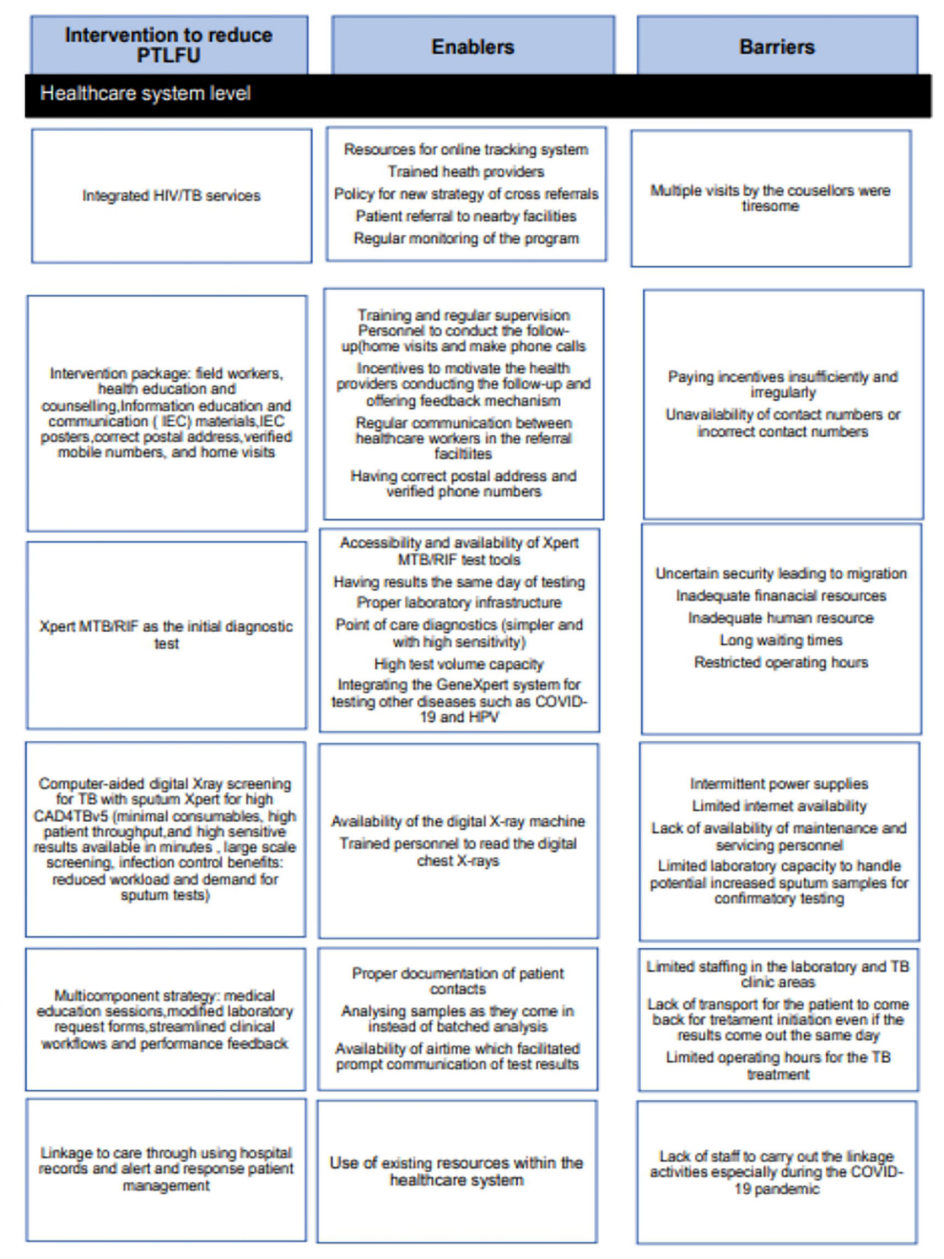
Interventions to reduce pre-treatment loss to follow-up, enablers and barriers at the healthcare system level.

We identified eight interventions from the included studies. We summarized the interventions together with enablers and barriers in Figures 2 and 3 and provided detailed information on enablers and barriers for the interventions in the Supplementary file 3 and Supplementary file 4 respectively.

### Interventions at the patient level

Five studies evaluated interventions at the patient level *.*

*Treatment support group*

A situation analysis in India assessed the effect of treatment support groups (TSGs) in providing information and access to quality health services to people with TB. ^46^ A TSG was defined as a ‘non-statutory body of socially responsible citizens and volunteers to provide social support to each needy TB patient safeguarding his dignity and confidentiality by ensuring access to information, free and quality services, and social welfare programs, empowering the patient for making decision to complete treatment successfully’. The TSG was meant to support people with TB from the time of diagnosis to the completion of treatment. The study found a decrease in the proportion of people experiencing PTLFU from 5% to 0% in the latest diagnostic cohorts. Success factors included high literacy rates in the community, strong political commitment, and human and financial resources. Challenges included lack of community advocacy for TSG services, lack of social inclusion standards, potential compromise of patient dignity and confidentiality and difficulties in holding individuals with diverse interests to the common goal of TB care.

*Mobile health interventions*

Three randomized controlled trials and a community-based TB prevalence survey evaluated mobile health (m-health) interventions. ^33, 36, 37, 48^ Two studies were conducted in India. One study found a 50% reduction in PTLFU and a reduction in delays in TB treatment initiation using telephone voice call reminders in addition to traditional referral letters and counselling compared to a control group that only received the referral letter and counselling. ^36^ The other study found WhatsApp messenger to be more effective than referral slips in communicating results during a community-based TB prevalence survey increasing patient registration for TB treatment from 55% to 100% and reduced the PTLFU from 45% to zero percent from the start to the end of the survey period. ^48^

The other two studies were conducted in South Africa. One study found that using short message service (SMS) to notify patients of their results was effective in initiating TB treatment compared to no SMS notifications; the SMS group saw a significantly shorter time to treatment initiation compared to the control group. ^37^ The other study randomized patients into three groups: 1) the control, people who did not receive any SMS reminder; 2) people who received a simple reminder to return to the clinic to collect their results; and 3) people who received a longer SMS reminding them to return to the clinic and that people die unnecessarily from TB because it can be cured. The results showed that sending an SMS the evening before the return date increased by 13% the likelihood of a patient returning to the clinic and start treatment, with the effect being larger in people living with HIV (39%). ^33^

The studies described several enablers for the m-health interventions: patients owning mobile phones with reliable up-to-date phone numbers, and be ready to register for the interventions; affordable calls and SMS charges; simple messages that do not reveal too much about the person; and training and financial support for the HCWs to deliver the intervention. A functional communication system with language preferences is also crucial. Barriers included phone battery depletion, power issues, phone sharing, and changing phone numbers which hindered timely message delivery. ^33, 36, 37^

### Interventions at the provider level

Two studies evaluated community-based interventions at the provider level. ^39, 45^ In a randomized trial conducted in Ethiopia, 12 communities received the intervention, where health workers from health centres collected sputum samples from people with presumptive TB and brought the samples to the diagnostic centre to be examined by microscopy and 20 communities did not. The trained community promoters distributed leaflets, discussed symptoms of TB during home visits and popular gatherings and encouraged symptomatic individuals to visit the outreach team or nearby health facility. After one year of the intervention, time to treatment decreased by 55% to 60% in the intervention group compared with 3% to 20% in the control group. The weighted mean duration differences between the intervention and control groups were statistically significant. ^39^ The second study in India evaluated a community-based active case-finding approach, where two TB reporting units received the intervention and the other two reporting units did not. The intervention comprised CHWs screening people for TB symptoms in the community, making referrals to government facilities, collecting sputum for transport to government laboratories, and supporting TB treatment. During the intervention, the number of people screened, referred, tested, provided test results, and initiated treatment were recorded and PTLFU decreased from 38% at baseline to 32%. ^45^

The studies described the following potential enablers: training and financial support (monthly stipend, travel, and communication allowance) for CHWs; having CHWs from the same community as patients to enhance acceptability and success of the intervention; engagement and involvement of community leaders; regular home visits; health education; actively tracking referrals; availability of diagnostic facilities for referral from the community; and monthly outreach clinics. However, they pointed out that in the facilities they could only access smear microscopy which has lower sensitivity for TB than molecular tests. ^39, 45^

### Interventions at the healthcare system level

Ten studies evaluated interventions at the healthcare system level *.*

*Multi-component intervention package*

Two studies utilized multicomponent strategies. ^41, 44^ The first study was conducted in India, which used a before and after design, measured the effectiveness of an intervention package that consisted of field workers who were recruited and posted at different medical colleges. The workers were responsible for offering counseling, health education and conducted follow-up visits for patients who did not attend the clinic or respond to phone calls. At the end of the 6-month intervention period, they compared number of referred people with TB initiated on treatment in peripheral health institutions and found that it improved from 46% at baseline to 66% and the difference was statistically significant. The key enablers included dedicated staff who conducted follow-up and were paid incentives for motivation. Additional enablers were training and regular supervision of health workers; having correct phone numbers and postal addresses for the follow-up visits and facilitating regular communication between the HCWs in the referral facilities. The factors that deterred the success of the intervention comprised insufficient or irregularly paid monetary incentives and incorrect or missing contact numbers for patient follow-up. ^41^

The second study, a Ugandan study, used before and after study design to evaluate the feasibility of a multifaceted intervention to improve treatment initiation among people with TB. The intervention involved medical education sessions for HCWs to understand the magnitude and consequences of PTLFU, modified laboratory request forms to collect detailed patient contact information, redesigned clinical workflow to improve sputum testing timeliness and providing desk phones to both the laboratory and outpatient department to enhance results dissemination. The study found that the median turnaround time for sputum test results improved from 12 hours to 4 hours post-intervention. The proportion of patients starting treatment within two weeks of diagnosis and those receiving same-day diagnosis and treatment initiation improved from 59% to 89% and 7.4% to 25%, respectively.

Key enablers for the intervention included proper documentation of patient contacts, analysis of samples as they come in, and availability of airtime on phones for prompt communication of test results. The study highlighted barriers such as limited staff in the laboratory and clinic areas, lack of transport for patients to come back to the facility for treatment initiation even if the results came out the same day, and limited TB treatment operating hours. ^44^

A Ugandan study evaluated the economic impact of a multicomponent strategy in a pragmatic cluster randomised trial. Participants were randomised into two groups: the intervention group, which used decentralized point of care molecular testing using the GeneXpert Edge platform, streamlined clinical work flows and performance feedback using monthly report cards, and the control group, which continued with routine care where sputum smear microscopy was performed onsite and molecular Xpert ^®^ MTB/RIF (Cepheid, Sunnyvale, CA, USA) performed at a centralised facility according to the national guidelines. The intervention strategy increased the number of patients diagnosed with TB and treated promptly, with a 10% increase in per-test cost of Xpert testing compared to routine care.

The high patient volume of about 500 people with TB annually made the strategy cost-effective. The GeneXpert system could also be used to test other diseases like COVID-19 and human papillomavirus. Having low patient volumes for TB testing were identified as a barrier. ^40^

*Use of Xpert MTB/RIF as the initial diagnostic test*

Two studies assessed Xpert MTB/RIF, an mWRD, as the initial diagnostic test for TB. ^34, 42^ In India, a before and after study compared the proportion of PTLFU during two time periods, one year before and one year after the introduction of Xpert MTB/RIF. In addition to Xpert MTB/RIF, the study utilized trained CHWs to identify people with TB symptoms and to help track patients. The study showed that the proportion of PTLFU decreased from 11% before the intervention to 4% after the intervention. ^42^ A cluster-randomized trial conducted in South Africa compared the effect on time to appropriate TB treatment initiation when GeneXpert was placed in a centralized sub-district level laboratory versus the clinic as a point-of-care (POC) test. The study found that the proportion of participants with culture-positive pulmonary TB initiated on appropriate TB treatment within 30 days was 76.5% in the laboratory arm and 79.5% in the POC arm, although the difference was not statistically significant. The median time to initiation of TB treatment was 7 days in the laboratory arm and 1 day in the POC arm. ^34^

For the strategy using Xpert MTB/RIF as the initial diagnostic test to work the following enablers were key: availability and accessibility of Xpert MTB/RIF cartridges and reagents; having results available on the same day of testing; infrastructure support (stable electricity supply, temperature control, adequate storage facilities); trained personnel and having simpler, more sensitive, and high-test volume capacity for POC diagnostics for economic feasibility. In the study where CHWs did follow-up, the following measures were in place to ensure that the Xpert MTB/RIF strategy worked: training every three months; a monthly stipend; involving CHWs from the same community as people with TB; and having the CHWs carry out monthly outreach in the villages. On the other hand, the barriers to the intervention were inadequate human and financial resources, long waiting times, and restricted operating hours. ^34, 42^

Two studies conducted an economic evaluation using Xpert MTB/RIF. ^38, 49^ The first study used data from a multi-centre trial (South Africa, Zambia, Zimbabwe, and Tanzania) to compare costs and clinical outcomes of POC Xpert MTB/RIF versus smear microscopy for diagnosing TB. The findings showed that while POC Xpert MTB/RIF might be more expensive than smear microscopy, it likely offered good value since it was associated with more people being started on treatment. ^38^ The second study, conducted in South Africa, used data from a pragmatic parallel cluster randomized trial. ^50^ The data were used to develop a mathematical model to explore the cost-effectiveness of the TB diagnostic algorithm. The findings suggested that the use of the algorithm in a high TB prevalence setting with a well-developed laboratory infrastructure might reduce PTLFU, although it would provide only minor reductions in mortality. However, larger benefits in health outcomes could be achieved by additional investment in the health system such as improving access to additional tests after a negative TB test. ^49^

*Integrated HIV/TB services*

One before and after study conducted in India tested the efficacy of a new model for delivering integrated HIV/TB services to improve the retention of co-infected individuals. At the end of the evaluation period, there was a 60% reduction in loss to follow-up and timely initiation of TB treatment. To enable this model to work, the study reported the following factors: having a policy in place that allows for cross-referrals; training health providers; tracking patients by telephone calls and home visits; referring patients to access TB and HIV services within the same health facility; and regular monitoring of the program. The authors proposed introducing an online tracking system and processes to decrease the multiple visits required by health providers. ^43^

*Hospital recording and an alert and response patient management*

A quasi-experimental study in South Africa evaluated the impact of linkage to care interventions on PTLFU in three provinces. The study assigned hospital recording and alert and response patient management interventions to each subdistrict. In hospital recording intervention, the existing routine referral mechanisms were utilized. The data clerks at each hospital identified newly diagnosed patients with TB and shared the information with the hospital staff to confirm treatment initiation. On the other hand, the alert-and-response patient management intervention identified all TB patients at selected primary healthcare facilities in addition to those identified in hospitals using routine data. The clerks in the hospital ensured patients not started on treatment after diagnosis were followed up by SMS, phone calls, and home visits by CHWs. The patients without telephonic information were immediately linked to CHWs. The study found a relative reduction in PTLFU in two provinces (42.4% and 22.3%) and no change in one province when you compare the baseline and intervention periods. The relative reduction was greater in subdistricts where alert and response patient management were implemented (34.2% to 49.3%) compared to subdistricts where hospital-recording was implemented (13.4%to32.2%). The key enabler to this intervention is that it was affordable given that it utilised the existing resources in the health system. The study highlighted limited staff to carry out linkage activities especially during the COVID-19 pandemic as a barrier. ^47^

*Computer-aided digital X-ray screening for TB with sputum Xpert MTB/RIF testing*

In Malawi, a three-arm randomized trial investigated the costs and yield of systematic HIV/TB screening using computer-aided digital chest X-ray (DCXR-CAD). ^35^ Participants were randomized to standard of care (SOC), health worker-directed HIV/TB screening; oral HIV testing and linkage to treatment (HIV screening), or oral HIV testing and linkage to treatment with additional digital chest X-ray screening for TB interpreted by computer-aided diagnosis software (CAD4TBv5 version 4.12.2 (Delft Imaging, the Netherlands) with sputum Xpert MTB/RIF testing for participants with CAD4TBv5 score above 45 (HIV/TB screening). ^35^ Participants were followed for 56 days to establish the time to TB treatment, missed TB and HIV diagnoses, and cost-effectiveness The median time to TB treatment initiation was shorter (1 day) in the HIV/TB screening arm compared to the SOC arm (11 days) and HIV screening arm (6 days).

Although not cost-effective at the end of eight weeks of follow-up, computer-aided TB screening improved the quality of life of people with TB symptoms. However, the intervention needs to be utilized in a setting with large-scale screening, digital X-ray machine availability, and trained personnel. On the other hand, having intermittent power supplies, limited internet availability, lack of availability of maintenance and servicing personnel, and limited laboratory capacity to handle potential increased sputum samples for confirmatory testing will be barriers for the intervention to work. ^35^

### Conceptual framework

**Figure 4.**
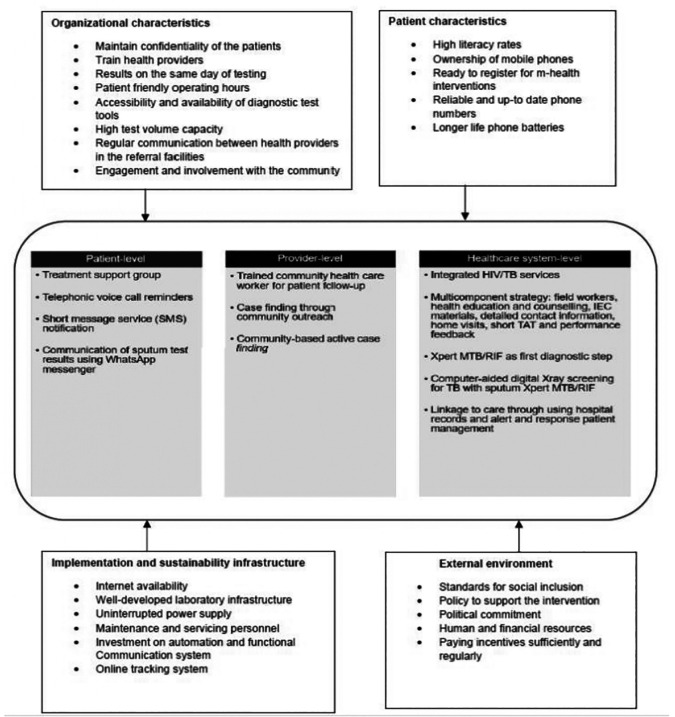
Conceptual framework on implementation considerations for the interventions to reduce pre-treatment loss to follow-up.

The framework ( Figure 4) describes practical options to minimize patient losses before starting treatment and may enable decision-makers to make recommendations applicable to their settings.

## Discussion

Our scoping review identified eight interventions from 17 quantitative studies from LMICs with one study utilizing a qualitative component. Most studies were randomized controlled trials (47%) or before and after interventions (24%). The interventions focused on offering people-centred care, such as through TSGs and timely test results, as well as prompt treatment initiation by using multi-component strategies, Xpert MTB/RIF as the initial diagnostic test, integrating TB/HIV care, active linkage to care, and leveraging the CHWs network. Although studies included in this review assessed the use of Xpert MTB/RIF, we note that it has now been superseded by Xpert MTB/RIF Ultra. Our review highlighted key enablers and barriers for implementing these interventions and proposed a framework emphasizing the need to consider patient characteristics, organizational factors, external influences, and infrastructure for successful implementation and sustainability.

Treatment support groups (TSGs) showed a reduction of PTLFU though this intervention was not tested in a randomized controlled trial. Patient support groups have been proposed as one of the ways of reducing TB-related stigma, a key contributor to PTLFU, and have been associated with better treatment outcomes for people with TB. ^51, 52^ Patient support groups have been suggested as one way of promoting the well-being of people with chronic illnesses apart from managing the disease. In a systematic review conducted by Thompson and colleagues, ^53^ the effectiveness was not clear though some studies reported positive health outcomes such as improvement in quality of life and clinical outcomes. Embuldeniya and colleagues conducted a qualitative synthesis and found that the positive impact of peer support interventions depends on how ‘peers’ are defined and also addresses the hierarchical aspects of the peer support intervention. ^54^ In this study, for the TSG to work, a high literacy rate from the community was required to facilitate capacity building of the support group; community advocacy and strong political commitment which enabled the operations of the support groups, standards for social inclusion, and human and financial resources for the sustainability of the support groups. Moreso, maintaining the dignity and confidentiality of the people with TB was considered so that mutual trust was developed in the TSG and maximum benefits were achieved. For this setting, the aforementioned factors promoted the success of the TSG although different settings need to conduct participatory action research to offer peer support interventions that reflect the local needs.

Mobile-health (m-health) interventions were reported in four studies. ^33, 36, 37, 48^ Three studies reported a shorter time from diagnosis to treatment initiation while one study reported reduction of PTLFU over a period of time. These interventions improve communication and coordination between healthcare workers and patients, addressing navigational challenges in TB care. ^55^ Although m-health is gaining popularity and showing positive health outcomes, most research has been conducted in high and upper-middle-income countries, raising questions about its applicability in low and lower-middle-income settings. ^56^ Studies found m-health interventions feasible due to affordable call and SMS costs. Key implementation considerations include mobile phone ownership rates, valid phone numbers, language preferences, confidentiality, staff training, minimizing phone sharing, and providing long-life batteries to ensure that users receive messages at an appropriate time. ^33, 36, 37^ These factors align with findings from two systematic reviews of barriers to m-health interventions in low and middle-income settings. ^57, 58^

Community-based interventions were reported in two studies. ^39, 45^ Both studies used CHWs to deliver the intervention, and the proportion of those who experienced PTLFU was reduced. Community-based interventions in mental health and child and adolescent health have also been found to be effective. ^59, 60^ Training of CHWs on TB and its management and offering them financial support (monthly stipend, monthly travel, and communication allowance) are enablers for effective delivery of the intervention. Recruiting CHWs from the same community as patients may enhance the acceptability and success of the intervention. Other important considerations are conducting regular home visits, providing health education that helps in understanding TB, ways of transmission and management, tracking referrals, increasing the availability of WHO-recommended rapid tests, and involving community leaders. ^39, 45^

Integration of TB/HIV services was reported to aid the timely initiation of TB treatment and thus reduce PTLFU. People with HIV are at higher risk for TB, which is a leading cause of death for them. ^61– 63^ Effective integration can decrease patient attrition by enabling quicker referrals and coordinated care. Recommended actions to enhance integration: incorporating policies that allow for cross-referrals; involving trained HCWs; tracking patients by phone calls and home visits; referring patients to TB and HIV services within the same health facility; and regularly monitoring the program. ^43, 64^ A systematic review by Legido-Quigley and colleagues found that integration of TB and HIV services benefited both providers and patients. However, the authors highlighted challenges such as the need for staff training, addressing the perceived association of TB and HIV, infrastructure improvements (such as a private space for HIV counselling and integrated records), and TB infection prevention and control. ^61^

In comparison to smear microscopy, Xpert MTB/RIF has been shown to reduce the time to initiation of treatment of TB and may be associated with a reduction in PTLFU. ^34, 42, 65^ Key implementation considerations for the use of Xpert MTB/RIF are investments by the National TB Program in improving the laboratory infrastructure by, for example, providing a stable source of electricity, temperature control, and adequate storage facilities. Additionally, when used at POC, Xpert MTB/RIF should be positioned where there is high test volume capacity and utilize trained personnel. ^34^

Many of our findings are aligned with the WHO recommendations on approaches and strategies for increasing access to rapid TB diagnostics as the initial test. In their report, the WHO emphasises person-centred care, near patient and same-day TB testing; support for patient costs (like transportation and food vouchers); dedicated staff training; improved data management systems; and integration of TB programs with other services such as HIV. ^66^

Although the included studies measured the effect of interventions on PTLFU statistically, the results were consistent with a reduction in the time to treatment duration with some of the interventions reducing up to one day. ^34, 35^ This implies that the TB treatment delay was averted. Treatment delay has been associated with clinical severity and ongoing TB transmission. ^67– 69^ Thus, prompt initiation of effective treatment would reduce clinical severity, continued TB transmission, drug resistance, and TB-related deaths, thereby improving the well-being and quality of life for people with TB. ^3– 5^

Of note, most of the intervention studies were conducted in India (n=7) and South Africa (n=6), both high TB burden settings. However, the findings may not apply to other high TB burden settings due to different contextual factors. Only one study included a qualitative component, limiting the insights into the acceptability and feasibility of these interventions and their implementation strategies. Also, we found no available evidence on patient navigators, people who assist patients to reach the next level of care in their TB journey, or on the electronic linkage of laboratory and treatment registers, which have been suggested as ways to reduce PTLFU in some settings. ^11, 70^ Apart from m-health interventions, Xpert MTB/RIF as an initial diagnostic test, multicomponent strategies, and community-based interventions had more than one study assessing their effect on on PTLFU, the other interventions were assessed in only one study, which limited our confidence in the study findings.

Our scoping review had several strengths. First, we conducted a comprehensive search in eight different databases and searched reference lists of included studies. Second, we performed double data extraction to ensure we captured all the relevant information to the review question. Third, we extracted information on key enablers and barriers to guide intervention implementation. Lastly, we reported our findings according to the PRISMA-ScR guidance.

Our review also had some limitations. We included studies published in English only and thus may have missed relevant studies written in other languages. In addition, we did not assess the risk of bias in the included studies, which could have influenced the internal validity of the findings, leading to an overestimation or underestimation of the actual benefit of the intervention. ^71^ Unlike systematic reviews, mandatory critical appraisal of included studies is not required for scoping reviews; nonetheless, we think a risk of bias assessment would have benefitted the review. ^72^ In addition, though considered to be an optional step in the framework, ^17, 18^ we were not able to have follow-up discussions with the relevant stakeholders to get information beyond our review findings.

## Conclusion

Our scoping review identified eight interventions that reduced PTLFU. The interventions targeted patients, providers, and healthcare system, though most addressed healthcare system-level challenges. The interventions reduced delays between diagnosis and treatment initiation by utilizing multi-component strategies, Xpert MTB/RIF as the initial diagnostic test, integrated HIV/TB care, CHWs, and mobile notifications. Despite these interventions reducing PTLFU, there was limited evidence on the effect of support groups and integration of HIV/TB care. Important implementation considerations such as user acceptability, infrastructure, resources, policies to guide the intervention implementation, and political commitment, are crucial before adopting the interventions given that healthcare environments vary. Our review identified only one study that had a qualitative arm that could provide more insight into the acceptability and feasibility of these interventions to inform effective approaches for intervention implementation. Future research should be people-centred and address social and economic factors that may affect PTLFU.

## Ethics and consent

For this review, we did not pursue a formal ethical review and consent since the data were published and publicly available. Ethical approval and consent were not required

## Author contributions

MM, EO, and TY gave input to the scoping review protocol. MM and EJO screened the studies. MM developed the data extraction tool, and all the authors gave their input before the data extraction process commenced. MM and EJO conducted data extraction. All authors gave their input in the description and synthesis of the data. MM wrote the first draft of the manuscript and all authors contributed to the writing of the manuscript.

## ORCID identifiers

Mercy Namuma Mulaku: 0000-0003-2480-0814

Eddy Johnson Owino: 0000-0001-9549-379X

Eleanor Ochodo: 0000-0002-7951-3030

Taryn Young: 0000-0003-2406-081X

## Data Availability

No data are associated with this article. OSF: Extended data for Interventions and implementation considerations for reducing pre-treatment loss to follow-up in adults with pulmonary tuberculosis: a scoping review. https://doi.org/10.17605/OSF.IO/YEKXQ. ^73^ The project contains the following underlying data:
1. PRISMA ScR checklist 2. Supplementary file 1: search strategy 3. Supplementary file 2: Excluded studies 4. Supplementary file 3: Enablers to interventions to reduce PTLFU 5. Supplementary file 4: Barriers to interventions to reduce PTLFU 6.
Table 1: Key characteristics of the included studies Data are available under the terms of the CC0 1.0 Universal

## Software availability statement

In this review computer-aided diagnosis software (CAD4TBv5 version 4.12.2 (Delft Imaging, the Netherlands)) has been used which is a proprietary software.

We provide details of an alternative free software that can perform an equivalent function:

3D slicer image computing platform version 4.11.0 https://www.slicer.org/ and https://pubmed.ncbi.nlm.nih.gov/22770690/.
