# Supplementary material for: Interventions and implementation considerations for reducing pre-treatment loss to follow-up in adults with pulmonary tuberculosis: A scoping review
Source: F1000Res. 2024 Nov 27;13:1436. [Version 1] doi: 10.12688/f1000research.157439.1 (PMC11733733; doi:10.12688/f1000research.157439.1)
Supplement: Peer review history

# Reviewer report: Reviewer response for version 1

**DOI:** 10.5256/f1000research.172883.r344162

**Published:** 2025-01-13

**Authors:** Hanlie Myburgh

**Affiliations:** 1 University of Amsterdam, Amsterdam, The Netherlands; 2 Desmond Tutu TB Centre, Stellenbosch University Faculty of Medicine and Health Sciences, Cape Town, Western Cape, South Africa

This review makes an important academic contribution by reviewing the available evidence on interventions to reduce PLTFU in adults with pulmonary TB worldwide. As the authors justify in their introduction, PLTFU is a major contributor to suboptimal TB programme performance and control efforts. The scoping review which they conducted is rigorous and they have clearly set out their aims/objectives, process/method, as well as inclusion/exclusion criteria, following specified frameworks (e.g., PCC and PRISMA-Sc). Well done on a very important and well written/presented review. I have only minor comments for consideration.  

 Results section: the reference on page 6’s ‘description of the interventions,’ to the eight interventions identified, is not clear in Figures 2 and 3. There are many more than 8 listed in the figure rows. For clarity, I suggest adapting the figures to identify the intervention areas more clearly, as well as in the text that follows, as the intervention under provider-level is not similarly italicized as in the other two levels.

 Figure 4, pages 11 and 12: I suggest refining/expanding the description of the conceptual framework/figure to better introduce and describe the 4 avenues/boxes identified (organizational characteristics, patient characteristics, implementation and sustainability infrastructure, and external environment) that impact on intervention implementation. While this is explained well in the discussion section, it is not equally apparent when looking at the figure and the caption, or the description of the conceptual framework at the end of the results section on page 11.

Are the rationale for, and objectives of, the Systematic Review clearly stated?

Yes

Is the statistical analysis and its interpretation appropriate?

Not applicable

If this is a Living Systematic Review, is the ‘living’ method appropriate and is the search schedule clearly defined and justified? (‘Living Systematic Review’ or a variation of this term should be included in the title.)

Not applicable

Are sufficient details of the methods and analysis provided to allow replication by others?

Yes

Are the conclusions drawn adequately supported by the results presented in the review?

Yes

Reviewer Expertise:

Tuberculosis and HIV, social aspects of disease, implementation science, health systems, qualitative research, scoping reviews

I confirm that I have read this submission and believe that I have an appropriate level of expertise to confirm that it is of an acceptable scientific standard.

# Reviewer report: Reviewer response for version 1

**DOI:** 10.5256/f1000research.172883.r347189

**Published:** 2024-12-31

**Authors:** Abhishek Sen

**Affiliations:** 1 Indian Institute of Public Health Gandhinagar, Gandhinagar, India

**Summary of the Article**

 The article titled "Interventions and implementation considerations for reducing pre-treatment loss to follow-up in adults with pulmonary tuberculosis: A scoping review" is a scoping review that addresses pre-treatment loss to follow-up in adults with pulmonary TB, a critical issue in TB management that exacerbates transmission and mortality rates. The authors aim to identify and evaluate interventions to reduce PTLFU, discuss barriers and enablers to their implementation, and propose a conceptual framework for intervention deployment.

**Key findings** **of the Article**

- **Interventions**: Eight interventions were identified, including treatment support groups (TSGs), mobile notifications, integrated HIV/TB services, and the use of advanced diagnostic tools like Xpert MTB/RIF.
- **Barriers**: Issues such as stigma, inadequate resources, and infrastructural challenges hinder effective implementation.
- **Enablers**: Mobile phone access, same-day TB testing, and community health worker (CHW) involvement emerged as facilitators.
- The authors developed a **framework **based on the Practical, Robust Implementation, and Sustainability Model (PRISM) to support context-sensitive intervention implementation.

**Strengths of the Article**

1. The review synthesizes evidence from multiple databases and diverse study designs, providing a holistic view of available interventions.
2. The conceptual framework offers practical guidance for policymakers and healthcare providers in tailoring interventions to specific contexts.
3. By identifying these factors, the article enhances the utility of interventions in real-world settings.
4. The use of double data extraction and adherence to PRISMA-ScR guidelines strengthens the reliability of findings.
5. The focus on low- and middle-income countries (LMICs), where TB burden is highest, ensures the article’s practical relevance to global health priorities.

**Weaknesses of the Article**

1. Only one study included qualitative data, which limits understanding of patient and provider perspectives on intervention feasibility.
2. The predominance of studies from India and South Africa may reduce the applicability of findings to other high-burden regions with different healthcare systems.
3. Interventions like patient navigators and electronic linkages, which have shown promise in other chronic conditions, are scarcely addressed.

I hope this gives you an idea about my understanding of the article.

Are the rationale for, and objectives of, the Systematic Review clearly stated?

Partly

Is the statistical analysis and its interpretation appropriate?

Not applicable

If this is a Living Systematic Review, is the ‘living’ method appropriate and is the search schedule clearly defined and justified? (‘Living Systematic Review’ or a variation of this term should be included in the title.)

Not applicable

Are sufficient details of the methods and analysis provided to allow replication by others?

Partly

Are the conclusions drawn adequately supported by the results presented in the review?

Yes

Reviewer Expertise:

Tuberculosis

I confirm that I have read this submission and believe that I have an appropriate level of expertise to confirm that it is of an acceptable scientific standard.

# Reviewer report: Reviewer response for version 1

**DOI:** 10.5256/f1000research.172883.r347193

**Published:** 2024-12-19

**Authors:** Vineet K Chadha

**Affiliations:** 1 National Tuberculosis Institute, Bengaluru, India

**Introduction**

**Authors have summarized the effects of various interventions undertaken in high TB burden settings to address PTLFU among adult Pulmonary TB patients and the barriers and enables thereof, using standard review methods. Using the synthesized evidence, they also developed a framework as a guide for reducing PTLFU. Therefore, this publication would be of great significance. It is very well written with good flow for easy understandability of health care workers and administrators at all levels.**

**Minor comments are as under:-**

- **Para 1: May update the data-figures as per the latest Global TB Report**
- **There is some repetition in 1 ^st^ para of ‘results” **
- **Treatment support Group: This evidence is from just one district of India, has to be stated accordingly. **
- **Likewise for other studies, the geographical level/target group in which evidence was generated has to be mentioned. **

Are the rationale for, and objectives of, the Systematic Review clearly stated?

Yes

Is the statistical analysis and its interpretation appropriate?

Not applicable

If this is a Living Systematic Review, is the ‘living’ method appropriate and is the search schedule clearly defined and justified? (‘Living Systematic Review’ or a variation of this term should be included in the title.)

Not applicable

Are sufficient details of the methods and analysis provided to allow replication by others?

Yes

Are the conclusions drawn adequately supported by the results presented in the review?

Yes

Reviewer Expertise:

Tuberculosis, Epidemiology

I confirm that I have read this submission and believe that I have an appropriate level of expertise to confirm that it is of an acceptable scientific standard.
